# First Assessment of Plasticizers in Marine Coastal Litter-Feeder Fauna in the Mediterranean Sea

**DOI:** 10.3390/toxics9020031

**Published:** 2021-02-04

**Authors:** Sabrina Lo Brutto, Davide Iaciofano, Vincenzo Lo Turco, Angela Giorgia Potortì, Rossana Rando, Vincenzo Arizza, Vita Di Stefano

**Affiliations:** 1 Department of Biological, Chemical, and Pharmaceutical Science and Technology (STEBICEF), University of Palermo, I-90123 Palermo, Italy; iaciofanodavide@gmail.com (D.I.); vincenzo.arizza@unipa.it (V.A.); vita.distefano@unipa.it (V.D.S.); 2 Department BioMorf, University of Messina, Viale Annunziata, Polo Universitario, 98168 Messina, Italy; vloturco@unime.it (V.L.T.); agpotorti@unime.it (A.G.P.); rrando@unime.it (R.R.)

**Keywords:** plastic pollution, marine litter, coastal areas, Crustacea Amphipoda, Mediterranean Sea

## Abstract

Micro and nanoplastics are harmful to marine life due to their high level of fragmentation and resistance to degradation. Over the past two decades, marine coastal sediment has shown an increasing amount of microplastics being a sort of trap for debris wastes or chemicals. In such an environment some species may be successful candidates to be used as monitors of environmental and health hazards and can be considered a mirror of threats of natural habitats. Such species play a key role in the food web of littoral systems since they are litter-feeders, and are prey for fishes or higher trophic level species. A preliminary investigation was conducted on five species of small-sized amphipod crustaceans, with the aim to understand if such an animal group may reflect the risk to ecosystems health in the central Mediterranean area, recently investigated for seawater and fish contamination. This study intended to gather data related to the accumulation of plasticizers in such coast dwelling fauna. In order to detect the possible presence of xenobiotics in amphipods, six analytes were scored (phthalic acid esters and non-phthalate plasticizers), identified and quantified by the gas chromatography mass spectrometry (GC-MS) method. The results showed that among all the monitored contaminants, DEP and DiBP represented the most abundant compounds in the selected amphipods. The amphipod crustaceans analyzed were a good tool to detect and monitor plasticizers, and further studies of these invertebrates will help in developing a more comprehensive knowledge of chemicals spreading over a geographical area. The results are herein presented as a starting point to develop baseline data of plasticizer pollution in the Mediterranean Sea.

## 1. Introduction

Marine sandy coastal areas are open ecosystems continuously influenced by factors such as tidal range, wave energy, and storm surges, which expose the sediment texture and faunal composition to potential sources of anthropogenic hazards [1,2]. This is particularly true for micro and nanoplastics that have a negative impact on marine life due to their high level of fragmentation and resistance to degradation [3]. Coastal sediment has shown that an increasing amount of microplastics has entered over the past two decades [4]. Such exposure to hazards influences the presence and the bioaccumulation of contaminants in organisms of the resident fauna [1,5], which may be successful candidates to be used as monitors of environmental and health hazards and can be considered a mirror of threats of marine coastal habitats [6]. This is particularly true for the sandy shores in Sicily, in the central Mediterranean Sea. The central position of the island, within the basin, makes the animal species inhabiting the coasts particularly useful for gathering extensive information on environmental features of the Mediterranean region, being a sort of trap for debris wastes or chemicals carried by the marine currents [5,7,8,9].

The coastal site investigated in this study is characterized by mobile sediment where biodiversity is not high and the faunal assemblage is mainly composed of the small-sized amphipod crustaceans belonging to the families Hyalidae Bulyčeva, 1957 and Talitridae Rafinesque, 1815. The members of these families, respectively marine and semi-terrestrial species, play an important role in the food chain of littoral ecosystems, such as beach debris feeders and prey for higher taxa [10,11]; their ecological role makes them good candidates for plastics assessment and monitoring in marine coastal areas.

The same geographical area was investigated regarding the spreading of PAEs (phthalic acid esters) and NPPs (non-phthalate plasticizers) in water samples and fishes collected from coastal sites in Tunisia [12]; thus an evaluation on the lower trophic levels was advisable to widen information on plasticizers occurrence. A previous study carried out by Baini et al. [13] showed a significant correlation between some plasticizers and microplastics in planktonic samples and suggested the use of plasticizer values detected in organisms as markers of microplastics exposure.

The microplastics are sources of plasticizers and others pollutants [14]; among plasticizers, the class of phthalates is the most important, and in fact they may constitute up to 50 per cent of the total weight of PVC (polyvinyl chloride) plastics [14]. Phthalates are released in the environment and occur in marine organisms that are in contact with these small molecules. There are few data on measured environmental concentrations of these compounds, mainly in marine sandy coastal areas. For instance, recent papers have detected chemical additives of microplastics in China [3] and in Italy [4]. While very few cases report the effects on the macrozoobenthos community under field conditions [15].

The main aims of this research were the assessment of the exposure to contaminants in target species and the comparison among the different species as potential sentinels exposed to marine swash and seagrass deposition. The data should be interpreted in order to offer preliminary data of environmental pollution that is potentially dangerous for marine life inhabiting the same area, and represents a possible threat throughout trophic species interactions of the food chain. It has been demonstrated that phthalates bioconcentrate in fishes [12,16] and can be differently metabolized among the species [17], resulting in a relevant danger when the fishes, or other edible animals, are not sensitive or vulnerable enough and become contaminants-carriers. Here, a preliminary study was carried out to obtain data on animals collected from the Mediterranean Sea and to observe the occurrence of plasticizers, PAEs and NPPs. The negative impacts of these pollutants shown to affect life traits make plasticizers detection a research priority under the guidelines for environmental quality proposed by the European Union [18].

## 2. Materials and Methods

### 2.1. Sampling

Sampling was carried out on the North-West coast of Sicily (Stagnone di Marsala locality, southern Italy), an area subjected to the main Mediterranean oceanographical current and close to different anthropogenic activities related to urban centres.

The locality is situated in the westernmost coast of Sicily, directly affected by the Atlantic Ionian Stream, which flows eastwards from the Atlantic Ocean through the Strait of Gibraltar [19]. The sites were micro-tidal and wave-dominated, with main currents from the west, and characterized by high variability of the substrate, from fine sand to cobbles and *Posidonia oceanica* banquette, reduced width and extension.

Pitfall traps were placed along an ideal transect perpendicular to the shoreline limit, with the trap zero (0 m) closest to the shoreline. Pitfalls, expected to catch surface-active individuals, were kept active for 24 h and emptied every twelve hours.

Two sampling events were conducted, June 2013 and May 2014, in correspondence with the most abundant population size, to collect a pool of individuals per species suitable for analyses.

A total of 426 individuals were collected and identified to species level. Females and juveniles, when collected with adult males, were included in species identification.

The five dominant amphipod species were selected: 23 specimens of *Talitrus saltator* (Montagu, 1808), 71 specimens of *Parhyale plumicornis* (Heller, 1886), 190 specimens of *Parhyale aquilina* (Costa, 1857), 73 specimens of *Speziorchestia stephenseni* (Cecchini, 1928), 69 specimens of *Orchestia montagui* Audouin, 1826 (Table 1). To perform the analyses, the sample size was higher in the smaller species, thus it was necessary to collect a greater number of *P. aquilina* than *T. saltator* or the other species.

### 2.2. Samples Extraction

The total specimens were separated into two pools (R1 and R2) per species containing both males, females and juveniles: *Talitrus saltator* specimens were subdivided into R1, composed of 11 specimens, and R2, composed of 12 specimens; *Parhyale plumicornis* in R1, 35 specimens, and R2, 36 specimens; *Parhyale aquilina* in R1, 95 specimens, and R2, 95 specimens; *Speziorchestia stephenseni* in R1, 36 specimens and R2, 37 specimens; *Orchestia montagui* in R1, 34 specimens, and R2, 35 specimens. The amphipods were dissected, and placed in cylindrical glass vials and stored at −20 °C.

Specimens were rinsed with distilled water once, then homogenized in 5 mL 99% EtOH and spiked with internal standards. After centrifugation (3 min, 1500× *g*), the supernatant was transferred to a 15 mL centrifuge tube prepackaged with 900 mg MgSO_4_ and 150 mg PSA (an ethylenediamine-N-propyl phase) for dispersive solid-phase extraction. The extracts were subjected to centrifugation (3 min, 1500× *g*), dehydrated through anhydrous Na_2_SO_4_ and concentrated to a volume of 0.5 mL with a constant flow of nitrogen. The obtained extracts were then analyzed by gas chromatography-mass spectrometry (GC-MS) for plasticizers determination.

### 2.3. Chemicals

Standards of phthalic acid esters (PAEs) and non phthalate plasticizers (NPPs), 99% purity, were purchased from Aldrich Chemical (Chicago, IL, USA). Internal deuterate standards of DBP-d4 and DEHP-d4 (at a concentration of 100 ng μL^−1^ in nonane), were purchased from Cambridge Isotope Laboratories Inc. (Andover, MA, USA). Ethyl acetate was used as a solvent for the preparation of individual stock and work solutions. The prepackaged 15 mL centrifuge tube with MgSO_4_ and PSA (ethylenediamine-N-propyl phase) were purchased from Sigma–Aldrich (Steinheim, Germany).

During sample preparation and analysis, no gloves or plastic material were used. Laboratory glassware was heated at 400 °C (for 4 h) and aluminium foil was used as a cover for minimizing the cross-contamination according to Fankhauser & Grob [20].

### 2.4. GC-MS Experimental Condition

The method proposed by Di Bella et al. [21] was used for plasticizers determination. A gas chromatograph (GC-2010) equipped with a quadrupole mass spectrometer (QP-2010 Plus) purchased by Shimadzu Italia (Milan, Italy) was employed. A capillary column (Supelco SPB-5MS; 30 m, 0.25 mm i.d., 0.25 µm film thickness) was used for separation of analytes in the following programmed temperature: from 60 to 190 °C with a gradient of 8 °C/min, 5 min hold at 190 °C, from 190 to 240 °C a gradient of 8 °C/min, 5 min hold at 240 °C, from 240 to 315 °C with a gradient of 8 °C/min. The carrier gas was helium (5.5 purity; constant rate of 30 cm/s); the transfer line temperature was 280 °C; the injector temperature was 250 °C. Injections were performed with a splitless injector, closed for 60 s, and then with a split ratio of 1:15. The acquisition was performed from 40 to 400 *m*/*z* in full scan and in Single Ion Monitoring (with ionization energy at 70 eV and emission current at 250 µA). As reported in Table 2, one target ion (T) and the two qualitative ions (Q1 and Q2) were used for each analyte.

## 3. Results

### 3.1. Quality Control, Calibration Curves, Linearity, Sensitivity, Repeatability and Recovery

Each standard solution was injected five times for the construction of the calibration curves. For quantitative analyses, the normalization of the areas was applied: for compounds with a retention time between 10.1 and 27.3 min, it was applied against the peak area of a characteristic fragment (*m*/*z* 153) of DBP-d4, whereas for compounds with a retention time between 30.2 and 38.2 min DEHP-d4, it was applied against the peak area of a characteristic fragment of DBP-d4 (*m*/*z* 153). The linearity, evaluated by R^2^ coefficient listed in Table 3, was good because it was better than 0.9885, except for DEHT, which had a value of 0.9802. The detection limits of (LOD (mg/kg) = 3 × RSD% × concentration) and of quantifications (LOQ (mg/kg) = 10 × RSD% × concentration) for each compound were calculated from the relative standard deviation percentage (RSD%) of six replicate injections at the lowest detectable concentration (with a signal-to-noise ratio <3). Results are reported in Table 3, and the values varied from 0.005 to 1.4 mg/kg for LOD and from 0.017 to 4.2 mg/kg for LOQ. At the lowest detectable concentration of each analyte, the RSD% values of peak area measurements (*n* = 6) were used to determine the repeatability. As can be seen in Table 3, the value was always better than 7.20%.

For recovery studies, the appropriate known amounts of each standard compound was added to a sample previously analyzed. After 24 h, the spiked sample was subjected to the pre-treatment procedures previously described. Recovery, calculated on the average of three replicate analyses, was between 83.2 and 111.3%.

### 3.2. Occurrence of PAEs and NPPs in Samples

Among the 19 compounds investigated, four PAE congeners (namely, DEP, DBP, DiBP and DEHP) and two NPPs (namely, DEHA and DEHT) were identified and quantified by GC-MS as described above. Table 4 shows their concentration and detection frequency in the species collected along the coastal environment of Sicily. As shown in Table 4, the mean value of ΣPAEs was 0.065 mg/kg and the mean value of ΣNPPs was 0.050 mg/kg in the specimens collected. Regarding the single species, *P. plumicornis* showed the highest level of concentration of PAEs respectively DEP 0.230 mg/kg; DiBP 0.240 mg/kg; DBP 0.046 mg/kg; and DEHP 0.066 mg/kg, and *S. stephenseni* (0.029 mg/kg DEP) and *P. aquilina* (0.027 mg Kg^−1^ DiBP; 0.013 mg/kg DBP; and 0.015 mg/kg DEHP) showed the lowest value of concentration in PAEs. Concerning the NPPs, the presence of DEHA was detected in all species; the highest value was detected in *O. montagui* (0.086 mg/kg) and the lowest value in *P. aquilina* (0.009 mg/kg), whereas the DEHT was detected only in *P. plumicornis* (0.335 mg/kg). Among the PAEs, DEP and DiBP represented the most concentrated compounds detected in the animals (max level detected 0.230 mg/kg and 0.240 respectively mg/kg), and DBP was the less concentrated compound (0.046 mg/kg).

## 4. Discussion

Plasticizers represent the main additives of plastic products and have been recognized as a cause of toxicity in aquatic and terrestrial organisms and the human population. Their occurrence in ubiquitous animal species is advisable to monitor wide geographical areas, in particular the marine coastal environment.

Among the aquatic and semi-terrestrial coastal taxa, amphipods have been considered sentinel species in several ecotoxicological studies; the talitrid species have been much more explored than the hyalid species. The *Orchestia* species have been proposed as candidates for the assessment of heavy metal toxicity in coastal ecosystems [22,23] and a huge literature on the *Hyalella* genus exists, where the species are recommended to estimate the toxicity of the sediments [24] or water [25]. In general, they are animals that can provide early warning signs of potential environmental risks, and can be used to monitor and prevent adverse health consequences [26].

However, a large portion of ecotoxicological studies has been performed in experimental laboratory conditions [27] where the water where the animals were kept was artificially contaminated. Rare cases have discovered contaminants in such invertebrates collected directly in the field [28]. Despite the potential ecotoxicological impacts, the occurrence and levels of plasticizers have also been poorly investigated in vertebrates species collected in the field, such as fishes [12].

The present research has shown data of the occurrence of plasticizers from a set of amphipod species collected in coastal sampling sites of the central Mediterranean Sea, for the first time. It follows a recent finding of plasticizers in marine habitats of the same geographical area. Gugliandolo et al. [12] analyzed seawater and tissues of *Sparus aurata*, where the most abundant and frequently detected plasticizers were DBP, DiBP, DEHP, DEHT, demonstrating that such compounds can bioconcentrate in fishes and probably through the food chain.

The amount of plasticizers in the studied amphipods is lower than the ones detected in fishes [12], as the latter represent a higher trophic level species, and with a longer lifetime. Coastal amphipods could ingest plasticizers, or absorb them directly, through microplastics or the organic fraction of sediment. Previous studies have observed the ingestion of microplastics by amphipods and the successive egestion after different intervals of post-feeding time [27,28,29]. Amphipods eliminate microplastics at different rates depending on different factors: 1. the shape and the size of the ingested particles, beads or fibres; 2. the concentration of microplastics in the food, thus in the environment; 3. the rate of feeding, where the faster the feeding, the more rapid the elimination of microplastics [27,28,29]. Consequently, it can be assumed that the gut passage of microplastics depends on diverse conditions. In lab experiments, *Gammarus fossarum* and *Echinogammarus marinus* egested microplastics after 16–48 h [29,30], while *Talitrus saltator* showed a complete elimination after a week [31].

The accumulation of microplastics in the gastrointestinal tract could be expected to be proportional to the translocation of plasticizers in the body of the amphipods; a hypothesis, still unclear in many aquatic invertebrates, that should be verified by studies on the metabolic pathways.

In the present study, the highest average values of PAEs concentrations were found in *Parhyale plumicornis* (DEP, DiBP, DEHP) and *Talitrus saltator* (DEP); the highest average concentrations of NPPs were found in *Orchestia montagui* (DEHA) and *Parhyale plumicornis* (DEHT).

Following data by Ugolini et al. [31], which observed a long time of retention of microplastics in the digestive tract of *Talitrus saltator*, we found that this species had more plasticizers (DEP, DiBP, DEHA, DEHP) than others.

*Parhyale aquilina* did not show the same chemical contamination as the other species, maybe due to high sensitivity. As shown in the literature, the toxicity of plasticizers showed a variable response in amphipods. Thuren & Woin [32] reported the effects of DBP detected in decreased locomotor activity in *Gammarus*. Call et al. [33] showed a different degree of toxicity in *Hyalella azteca*, related to different exposure concentration of the high molecular weight phthalates DHP and DEHP. A next step should be to determine the sensitivity of the various species to plasticizers, as a factor to take into account in selecting candidate sentinels of plastics-pollution.

In a general view, the plasticizers detected should be viewed as components of the whole ecosystem, and in the light of the different kinds of biological interactions between the amphipod species and co-inhabiting coastal fauna. The most relevant interaction regards the connections in the food web. It is noteworthy to remember that amphipods are the primary consumers in the marine environment, and can cause bioaccumulation in higher taxa, being preys to a wide range of animal groups from flatworms [34] to cetaceans [35].

There are many advantages to the use of amphipods as sentinel species in ecotoxicological studies. They are easy to capture, have a short generation turnover and are cosmopolitan species. The order Amphipoda is dominant in temperate coastal environments; most species are highly specific for a single habitat and display limited dispersal, thus exhibiting a strong genetic cohesion and taxonomic homogeneity [10,11,36,37,38], which provide spotted information on a regional spatial scale. Further, at the taxonomic level of genus, they are commonly found worldwide and analyses from different localities can be comparable as the co-generic species are ecological equivalents.

In summary, in this study, different detection patterns of plasticizers were drawn from various species of amphipods. The amphipod crustaceans analyzed were found to be a promising tool to detect and monitor plasticizers, and assessment of these chemicals will help in developing a more comprehensive knowledge of their spread over a geographical area. There are few results obtained from invertebrates directly collected from coastal marine habitat; this is a significant gap, which the present article can help to fill.

## Figures and Tables

**Table 1:** Sampling data: list of species, habitat, locality and coordinates, date, number of individuals per age and respective mean wet weight of the pool of individuals.

Species	Maturity Stage	Habitat	Locality	Coordinates	Date of Sampling Month/Year	Number of Individuals	Wet Weight mg, Mean ± SD
*P.plumicornis*	male	Sandy and rocky	Stagnone di Marsala	37°55.197′ N 12°28.214′ E	June 2013	14	21.82 ± 5.15
*P.plumicornis*	female	Sandy and rocky	Stagnone di Marsala	37°55.197′ N 12°28.214′ E	June 2013	25	7.66 ± 2.22
*P.plumicornis*	juvenile	Sandy and rocky	Stagnone di Marsala	37°55.197′ N 12°28.214′ E	June 2013	32	3.25 ± 1.19
*O.montagui*	male	*Posidonia* Banquette	Stagnone di Marsala	37°55.330′ N, 12°28.011′ E	June 2013	29	32.98 ± 5.38
*O.montagui*	female	*Posidonia* Banquette	Stagnone di Marsala	37°55.330′ N, 12°28.011′ E	June 2013	27	16.57 ± 5.35
*O.montagui*	juvenile	*Posidonia* Banquette	Stagnone di Marsala	37°55.330′ N, 12°28.011′ E	June 2013	13	2.74 ± 1.17
*S.stephenseni*	male	*Posidonia* Banquette	Stagnone di Marsala	37°55.330′ N, 12°28.011′ E	May 2013	13	29.54 ± 5.60
*S.stephenseni*	female	*Posidonia* Banquette	Stagnone di Marsala	37°55.330′ N, 12°28.011′ E	May 2013	33	24.14 ± 13.37
*S.stephenseni*	juvenile	*Posidonia* Banquette	Stagnone di Marsala	37°55.330′ N, 12°28.011′ E	May 2013	27	8.50 ± 3.45
*T.saltator*	male	Sandy	Stagnone di Marsala	37°55.330′ N, 12°28.011′ E	June 2014	9	68.85 ± 7.66
*T.saltator*	female	Sandy	Stagnone di Marsala	37°55.330′ N, 12°28.011′ E	June 2014	7	38.85 ± 3.58
*T.saltator*	juvenile	Sandy	Stagnone di Marsala	37°55.330′ N, 12°28.011′ E	June 2014	7	27.40 ± 1.13
*P.aquilina*	male	Sandy and rocky	Stagnone di Marsala	37°55.197′ N 12°28.214′ E	May 2014	64	5.38 ± 0.96
*P.aquilina*	female	Sandy and rocky	Stagnone di Marsala	37°55.197′ N 12°28.214′ E	May 2014	72	3.12 ± 0.69
*P.aquilina*	juvenile	Sandy and rocky	Stagnone di Marsala	37°55.197′ N 12°28.214′ E	May 2014	54	0.98 ± 0.41

**Table 2:** GC-MS data of analyzed plasticizers and BPA.

Compound	MW	Rt min.	T [*m*/*z* (%)]	Q_1_ e Q_2_ [*m*/*z* (%)]	
DMA	174	10.1	114 (100)	101 (85.1)	111 (78.1)
DEA	202	12.5	111 (100)	157 (87.4)	128 (65.7)
DMP	194	13.7	163 (100)	92 (10.3)	164 (10.0)
DEP	222	15.8	149 (100)	177 (25.7)	176 (12.3)
DiBA	258	17.1	129 (100)	185 (45.3)	111 (33.5)
DBA	258	18.6	129 (100)	185 (84.9)	111 (63.6)
DPrP	250	18.8	149 (100)	150 (9.2)	209 (7.5)
BB	212	19.0	105 (100)	91 (51.0)	212 (26.3)
DiBP	278	20.9	149 (100)	150 (9.4)	223 (8.3)
DBP	278	23.3	149 (100)	150 (9.0)	223 (5.7)
BPA	228	27.3	213 (100)	119 (17.4)	228 (17.4)
BBP	312	30.2	149 (100)	91 (74.6)	206 (29.1)
DEHA	370	30.9	129 (100)	112 (30.6)	147 (25.4)
DiHepP	362	31.9	149 (100)	99 (25.0)	265 (19.6)
DcHexP	330	33.8	149 (100)	167 (35.2)	150 (14.7)
DEHP	390	34.1	149 (100)	167 (37.9)	279 (15.9)
DPhP	318	34.4	225 (100)	226 (15.9)	104 (10.1)
DEHT	390	37.5	149 (100)	112 (81.8)	261 (59.4)
DEHS	426	38.2	185 (100)	149 (89.4)	112 (30.1)

MW, Molecular Weight; Rt, Retention time; T, target ion. Q1 and Q2, qualitative ions. Abbreviations: DMA, di-methyladipate; DEA, di-ethyladipate; DMP, di-methylphthalate; DEP, di-ethylphthalate; DiBA, di-(2-methylpropyl)adipate; DBA, di-n-butyladipate; DPrP, di-propylphthalate; BB, benzylbenzoate; DiBP, di-(2-methylpropyl)phthalate; DBP, di-butylphthalate; BPA, bisphenol A; BBP, benzylbutylphthalate; DEHA, di-(2-ethylhexyl)adipate; DiHepP, di-n-heptylphthalate; DcHexP, di-cyclo-hexylphthalate; DEHP, di-(2-ethylhexyl)phthalate; DPhP, di-phenylphthalate; DEHT, di-(2-ethylhexyl)terephthalate; DEHS, di-(2-ethylhexyl)sebacate.

**Table 3:** Linearity, sensitivity, repeatability and recovery.

Compound	R^2^	LOD (mg/kg)	LOQ (mg/kg)	RSD (%)	Recovery (%)
DMA	0.9933	0.010	0.030	2.22	111.3
DEA	0.9911	0.011	0.037	3.25	105.4
DMP	0.9954	0.005	0.023	4.21	102.5
DEP	0.9911	0.010	0.038	3.25	108.6
DiBA	0.9977	0.010	0.027	6.20	100.3
DBA	0. 9885	0.022	0.068	2.23	99.2
DPrP	0.9917	0.005	0.020	2.68	95.3
BB	0.9885	0.012	0.033	3.65	96.5
DiBP	0.9933	0.011	0.027	4.58	98.4
DBP	0.9941	0.007	0.016	5.69	101.4
BPA	0.9903	0.032	1.0	4.56	91.2
BBP	0.9822	0.042	0.121	7.20	105.7
DEHA	0.9877	0.030	0.09	6.66	99.5
DiHepP	0.9888	0.23	0.55	7.01	83.2
DcHexP	0.9952	0.031	0.087	4.33	89.5
DEHP	0.9999	0.005	0.016	2.56	107.7
DPhP	0.9941	0.016	0.051	2.58	94.5
DEHT	0.9802	0.077	0.233	1.96	105.4
DEHS	0.9941	0.022	0.053	3.33	102.5

**Table 4:** Plasticizer concentrations detected in the amphipod species.

Specimens Pools per Species		PLASTICISERS (mg/kg)					
		PAEs				NPPs	
Species	Replicate Pools	DEP	DiBP	DBP	DEHP	DEHA	DEHT
*T. saltator*	R1	0.168	0.099	0.029	0.059	0.023	nd
*T. saltator*	R2	0.114	0.049	0.022	0.051	0.019	nd
*P. plumicornis*	R1	0.230	0.240	0.046	0.300 *	0.031	0.335
*P. plumicornis*	R2	0.038	0.223	0.029	0.066	0.018	nd
*P. aquilina*	R1	0.059	0.027	0.016	0.016	0.009	nd
*P. aquilina*	R2	nd	nd	0.013	0.015	0.009	nd
*S. stephenseni*	R1	0.029	0.047	0.018	0.060	0.017	nd
*S. stephenseni*	R2	0.115	0.044	0.022	0.065	0.017	nd
*O. montagui*	R1	nd	0.042	0.013	0.033	0.017	nd
*O. montagui*	R2	nd	0.105	0.023	0.178 *	0.086 *	nd
	mean	0.108	0.097	0.023	0.046	0.018	
	S.D. ±	0.066	0.076	0.009	0.019	0.006	

nd ≤ LOQ; the values reported for each sample group are subtracted from the control values reported in the last column. * values considered outliers and not included in the mean.

## Data Availability

Data is contained within the article.
